# Supplementary material for: A genome-scale map of DNA methylation turnover identifies site-specific dependencies of DNMT and TET activity
Source: Nat Commun. 2020 May 29;11:2680. doi: 10.1038/s41467-020-16354-x (PMC7260214; doi:10.1038/s41467-020-16354-x)
Supplement: Supplementary file 1 — Supplementary Information [file 41467_2020_16354_MOESM1_ESM.pdf]

Supplementary Figures for:

A genome-scale map of DNA methylation turnover identifies site-specific dependencies of DNMT and TET activity

Ginno, Gaidatzis, Feldmann et al.

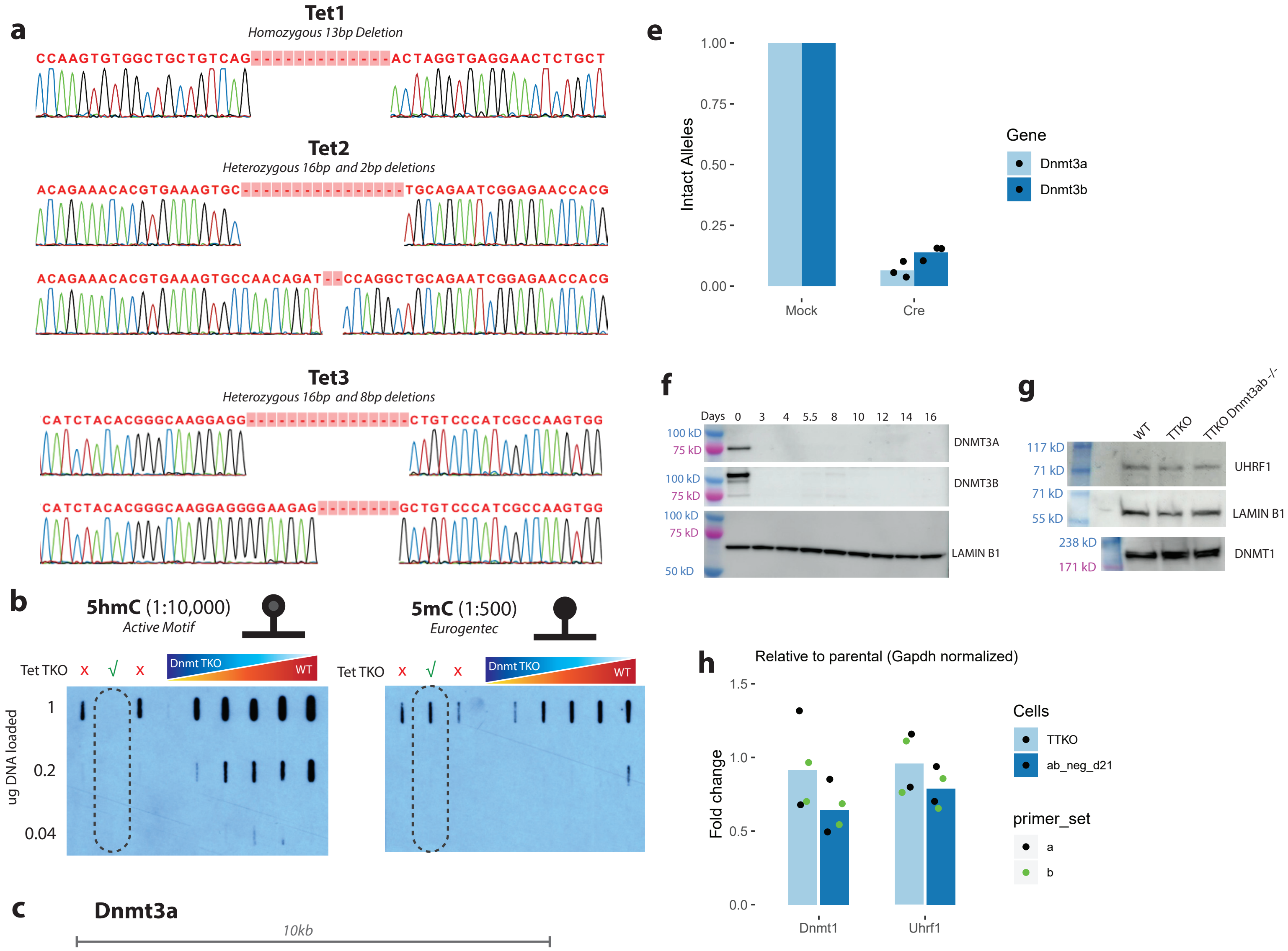

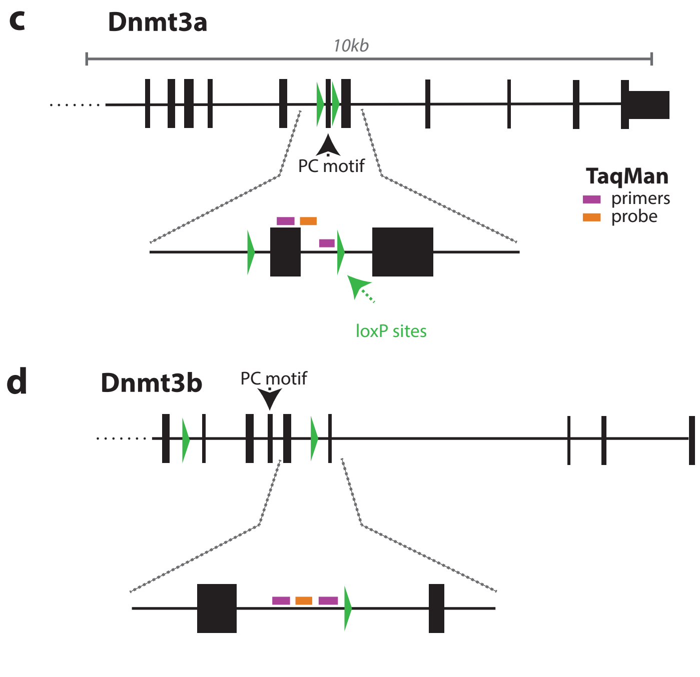

Gene	Forward primer	Reverse primer	Probe
Dnmt3a	GCAGAAGGTACCAGTTTAGAAAGCA	TGCCCGCAAGGGACTTTAT	AGGAGGGCACCTTAC
Dnmt3b	GCTGTGCAGGCAACATATGG	CCTTACGTGACCGAGCTGTCT	CAACTAACCGGAGGTTC
Gapdh	GAGCCCCAGGCTATCTCATG	GTTCTCCACACCTATGGTGCAA	TCTTCAGAGTGGAATACT

Supplementary Figure 1. Genotyping and characterization of cell lines. (a) Sanger sequencing confirmation of genetic deletion of the Tet enzymes. Genotype of deletion alleles is noted above the chromatograms for each Tet gene. (b) Slot blot of 5hmC (left) and 5mC (right) of tested lines. Lanes 1 and 3 represent clones where 5hmC signal was still present, while lane 2 is the line used in this study and is noted by a dashed oval. Lanes 4-9 show samples of Dnmt TKO DNA progressively mixed with wild-type DNA. (c,d) Genic models of loxP sites flanking catalytic exons for Dnmt3a and Dnmt3b respectively. Purple and orange bars represent primer positions and the probe used in TaqMan genotyping, respectively. The table below the gene graphics displays the primer sequences used for amplification and the probes used for Dnmt3a, Dnmt3b and Gapdh quantification in the TaqMan assay (e) Genotyping assay of triplicates at day 8 post Cre transduction for Dnmt3a (light blue) and Dnmt3b (dark blue). Shown are Dnmt levels relative to Gapdh signal. Bars represent mean levels relative to mock transduced cells and dots represent individual data points from triplicate measurements. (f) Western blot of DNMT3a, DNMT3b and LAMIN B1 as a loading control across noted days of the time course. All signals are from the same blot which was stripped and reblotted as described in methods. (g) Western blot of DNMT1, UHRF1 and LAMIN B1 as a loading control in different mutant backgrounds. TTKO Dnmt3a/b -/- refers to TTKO cells 21 days post Cre transduction. (h) RT-PCR measurements of Dnmt1 and Uhrf1 in TTKO and TTKO Dnmt3a/b -/- cells a in g. Values are normalized to Gapdh and fold change is relative to the parental line. Two PCR reactions for Dnmt1 and Uhrf1 are shown in duplicate and individual points are shown as black and green colored dots, while the bars represent the mean from all measurements.

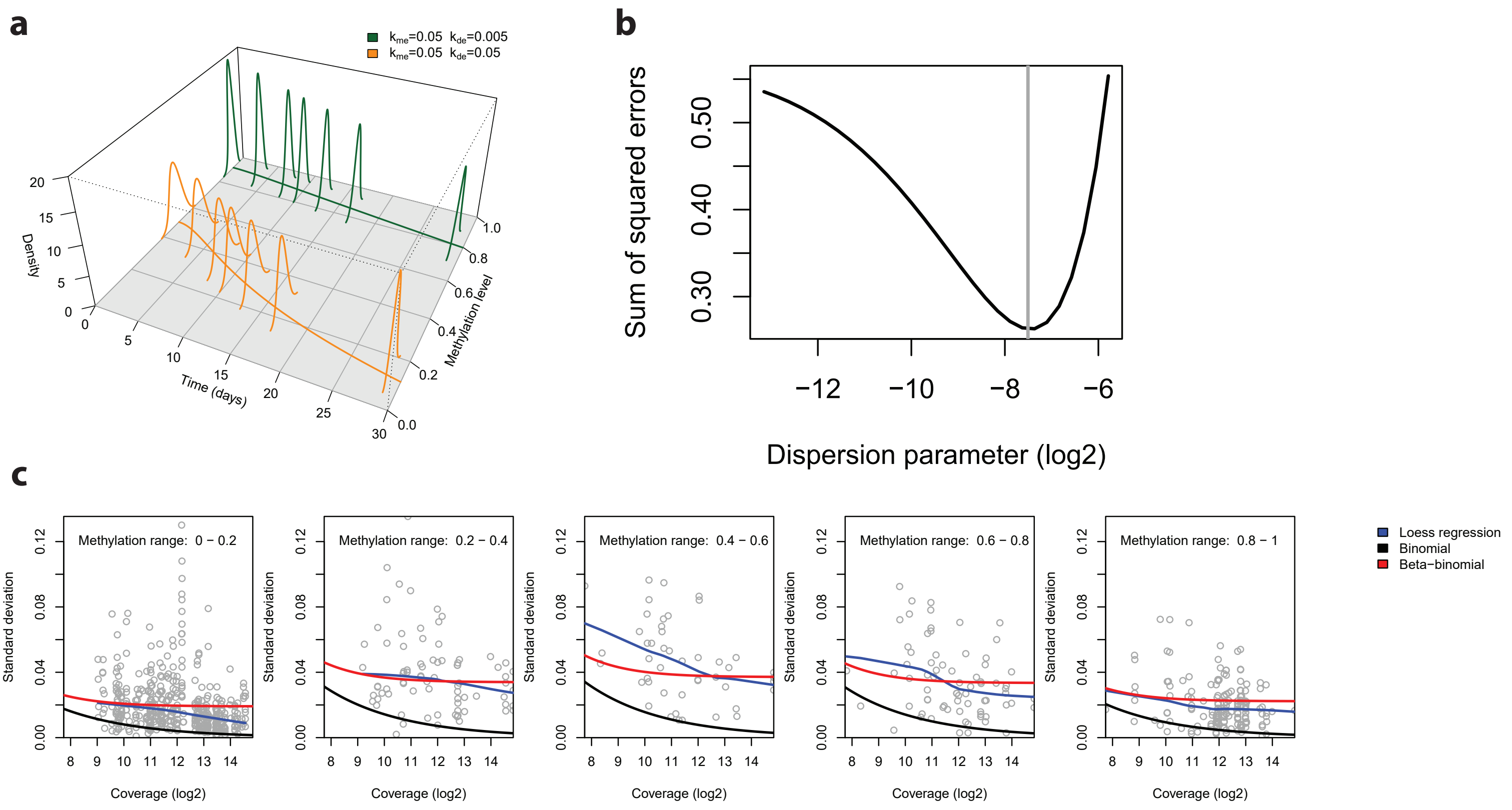

Supplementary Figure 2. Error model for confidence estimation of rate inference. (a) Error model for determination of confidence in rate inference. A beta-binomial distribution was calculated for methylation measurements at different steady states and Bayesian inference allowed determination of the rate likelihood given the data. The x-axis represents time in days, the y-axis represents steady state methylation levels, and the z-axis depicts density of methylation measurements for cytosines with the given average methylation level. The green line depicts decay kinetics for a highly methylated cytosine while the orange line represents decay for a 50% methylated cytosine. The legend indicates the most likely rates of methylation and demethylation for each cytosine given the fit between theoretical combinations and the observed decay. Note the increased variance in measurements for intermediately methylated cytosines. (b) Parameter sweep for determination of γ parameter in the beta-binomial. The minima in squared error was selected (grey line). (c) A beta-binomial best describes the error in methylation calls as a function of coverage. CpGs were binned on steady state methylation levels and coverage as a function of standard deviation for methylation calls is shown. Note, a beta-binomial (red line) more closely fits the relationship of standard deviation and coverage (Loess regression, blue line) than a binomial (black line).

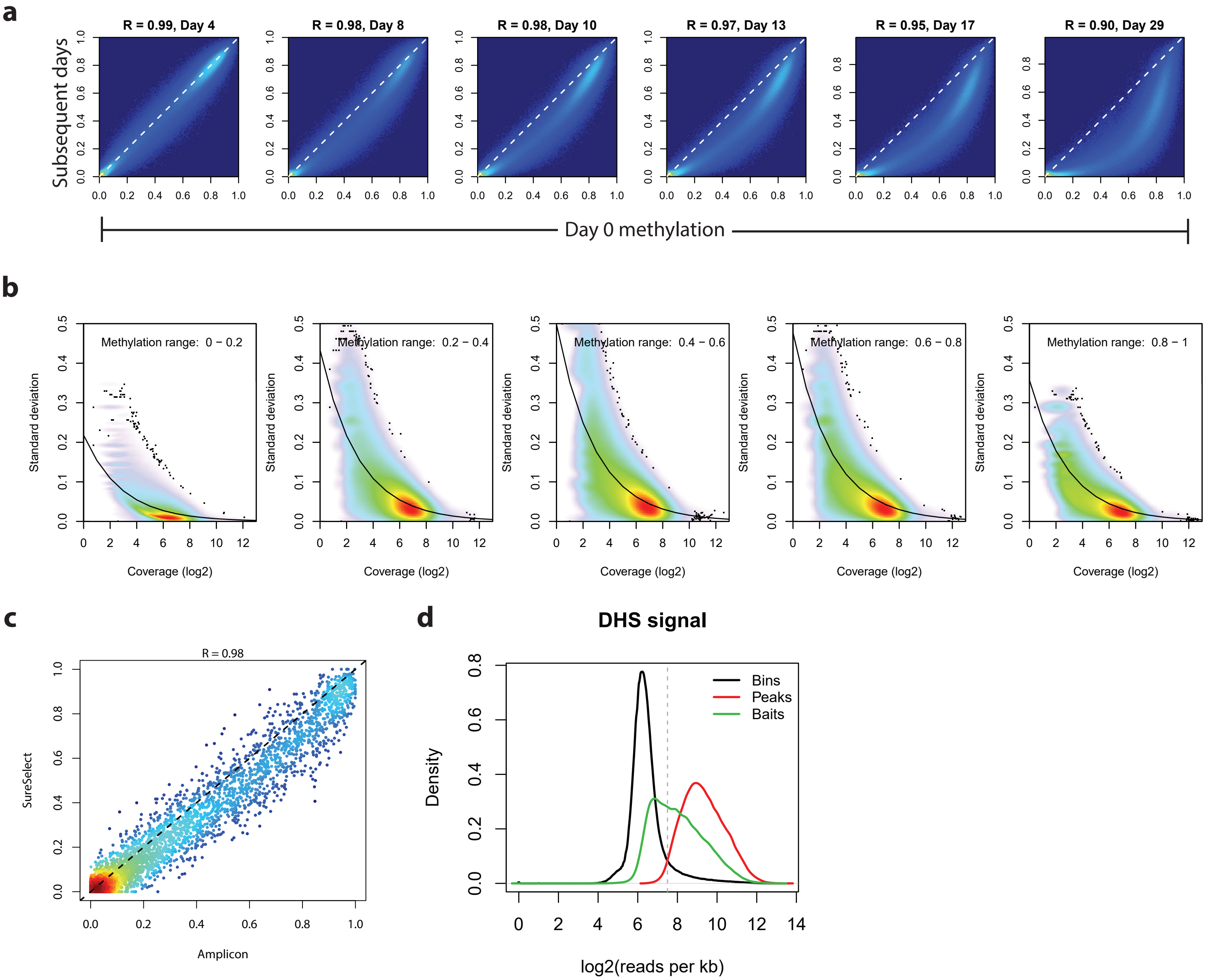

Supplementary Figure 3. Reproducibility and accuracy of SureSelect measurements. (a) Decay of methylation across the time course experiments. The x-axis for each plot represents Day 0, while the y-axes from left to right represent days, 4, 8,10,13,17 and 29 respectively. Note the progressive decay of methylation levels over time. (b) Variance in sampling methylation levels as a function of steady state for SureSelect experiments, as predicted using a binomial distribution. Methylation ranges are noted in the legend at the top left. The trend line represents predicted standard deviation as a function of the mean assuming random sampling from a binomial distribution. (c) Correlation of CpG methylation between amplicon and SureSelect measurements for CpGs assayed in both experiments (259 individual CpGs, 5439 measurements). The Pearson correlation coefficient is shown above the plot and axes represent proportion methylated molecules for each CpG at each time point in amplicon and SureSelect experiments. (d) Bait representation as a function of DHS signal. The mm9 genome was tiled into 1kb bins and mappable reads per kb tallied. The x-axis represents mappable reads/kb (minimum of 900bp per bin) and density on the y-axis depicts the distribution of bins at a particular coverage. Bins that overlap with DHS peaks are represented by the red line, note an increase in DHS signal/mappable kb for these regions. The green line represents DHS signal for bins covered in the SureSelect experiment. Note representation of both accessible and inaccessible sites. The grey line represents the cutoff between accessible and inaccessible regions, based on the difference in signal between genomic bins and bins overlapping DHS peaks.

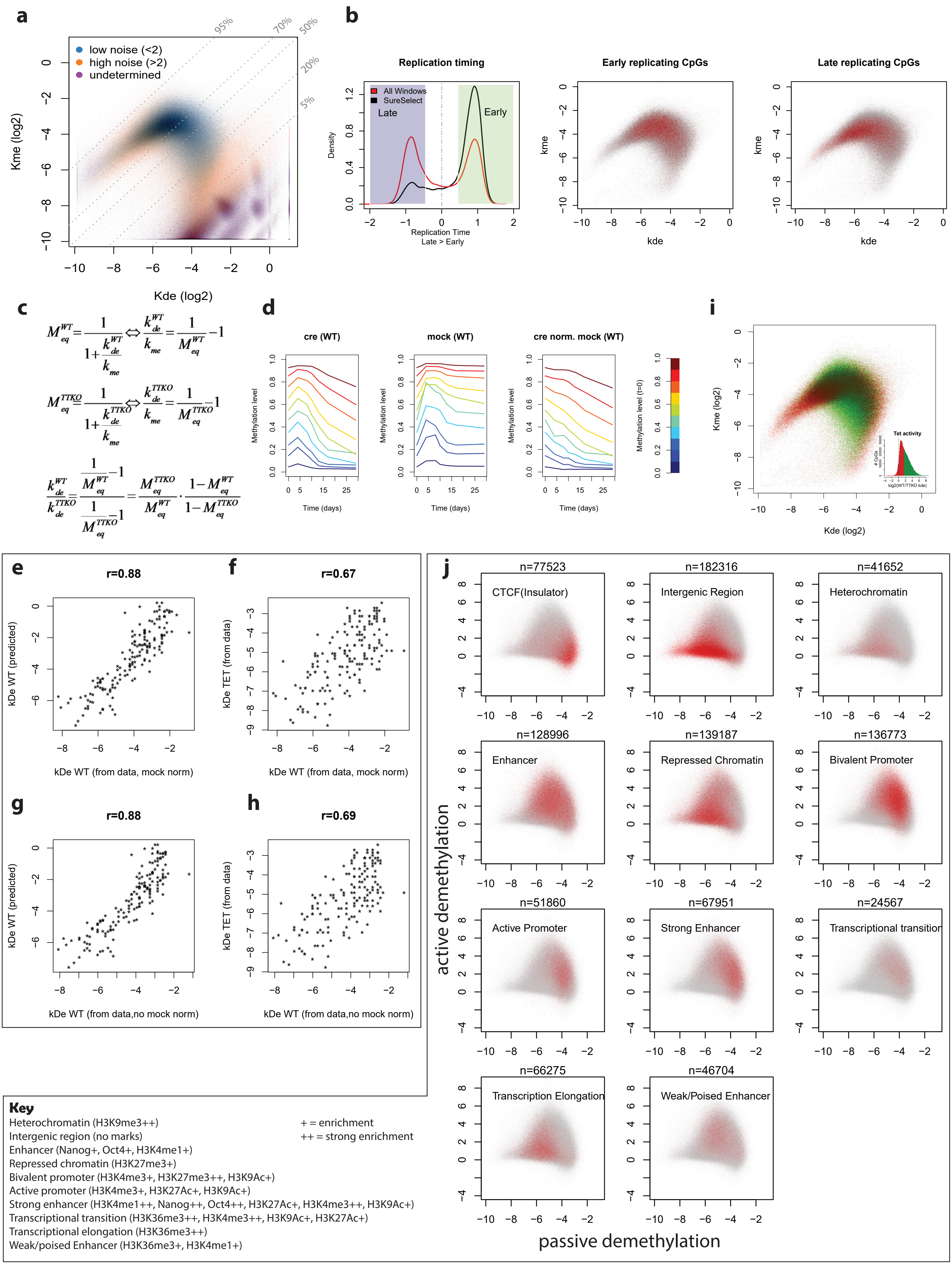
a
low noise (<2)
high noise (>2)
undetermined
95%
70%
50%
20%
5%
Kme (log2)
Kde (log2)
b
Replication timing
All Windows
SureSelect
Late
Early
Density
Replication Time
Late > Early
Early replicating CpGs
Late replicating CpGs
kme
kde
c
$M_{eq}^{WT} = \frac{1}{1+\frac{k_{de}^{WT}}{k_{me}}} \Leftrightarrow \frac{k_{de}^{WT}}{k_{me}} = \frac{1}{M_{eq}^{WT}} - 1$
$M_{eq}^{TTKO} = \frac{1}{1+\frac{k_{de}^{TTKO}}{k_{me}}} \Leftrightarrow \frac{k_{de}^{TTKO}}{k_{me}} = \frac{1}{M_{eq}^{TTKO}} - 1$
$\frac{k_{de}^{WT}}{k_{de}^{TTKO}} = \frac{\frac{1}{M_{eq}^{WT}} - 1}{\frac{1}{M_{eq}^{TTKO}} - 1} = \frac{M_{eq}^{TTKO}}{M_{eq}^{WT}} \cdot \frac{1-M_{eq}^{WT}}{1-M_{eq}^{TTKO}}$
d
cre (WT)
mock (WT)
cre norm. mock (WT)
Methylation level
Time (days)
Methylation level (t=0)
i
Kme (log2)
Kde (log2)
Tet activity
# CpGs
log2(WT/TTKO kde)
e
r=0.88
kDe WT (predicted)
kDe WT (from data, mock norm)
f
r=0.67
kDe TET (from data)
kDe WT (from data, mock norm)
g
r=0.88
kDe WT (predicted)
kDe WT (from data,no mock norm)
h
r=0.69
kDe TET (from data)
kDe WT (from data,no mock norm)
j
n=77523
CTCF(Insulator)
n=182316
Intergenic Region
n=41652
Heterochromatin
n=128996
Enhancer
n=139187
Repressed Chromatin
n=136773
Bivalent Promoter
n=51860
Active Promoter
n=67951
Strong Enhancer
n=24567
Transcriptional transition
n=66275
Transcription Elongation
n=46704
Weak/Poised Enhancer
active demethylation
passive demethylation
Key
Heterochromatin (H3K9me3++)
Intergenic region (no marks)
Enhancer (Nanog+, Oct4+, H3K4me1+)
Repressed chromatin (H3K27me3+)
Bivalent promoter (H3K4me3+, H3K27me3++, H3K9Ac+)
Active promoter (H3K4me3+, H3K27Ac+, H3K9Ac+)
Strong enhancer (H3K4me1++, Nanog++, Oct4++, H3K27Ac+, H3K4me3++, H3K9Ac+)
Transcriptional transition (H3K36me3++, H3K4me3++, H3K9Ac+, H3K27Ac+)
Transcriptional elongation (H3K36me3++)
Weak/poised Enhancer (H3K36me3+, H3K4me1+)
+ = enrichment
++ = strong enrichment

Supplementary Figure 4. Confidence in rate inference and genomic demethylation context. (a) Scatterplot of k_{me} and k_{de} for all cytosines in the experiment with sufficient coverage. Points are colored based on confidence for rate inference, blue being low noise, orange high noise, and purple is undetermined (see methods for confidence determination). (b) Relationship between replication timing and methylation/demethylation rates (see methods for replication data). Left panel: density distribution of replication timing with the red line reflecting all 5kb genomic windows and the black line reflecting windows with SureSelect representation. The light blue box denotes late replicating regions, while the green box denotes early replicating regions. Middle panel: Early replicating CpGs (red points) reveal a shift towards the right and lower side of the scatter and represent more euchromatic representation. Right panel: CpGs with higher methylation (red points) and reduced turnover tend to overlap with later replicating regions. Number of CpGs in early replicating regions were randomly subsampled to the number of late replicating CpGs. (c) Equation (3) to estimate change in the demethylation rate between WT and TTKO cells. Meq represents methylation level at equilibrium, while superscripts of WT and TTKO represent wild-type and Tet Triple KnockOut respectively. All other symbols are as portrayed in Fig. 1a, g.
(d) Methylation time course measured by amplicon sequencing in wild-type cells. CpGs were binned based on starting methylation in 10% increments, and the mean decay over triplicates for Cre transduced samples (left), mock samples (middle), and transduced samples after mock normalization (right). (e) Accuracy of k_{de}^{WT} prediction by only using difference between TTKO and WT methylation levels at steady state (see panel c). The x-axis depicts kde^{WT} inferred from the time course, while the y-axis shows predicted k_{de}^{WT}. Methylation levels were normalized to mock samples (see methods). (f) Correlation between kde^{WT} and kde^{TTKO} inferred from the data. (g) Accuracy of k_{de}^{WT} prediction as in panel e but without normalization to mock samples. (h) Correlation between k_{de}^{TTKO} and k_{de}^{WT} as in panel f but without mock normalization. (i) Rates with high confidence inference for individual CpGs in TTKO cells colored by TET activity. Red points represent CpGs with low TET demethylation activity while demethylation rates at CpGs denoted in green are affected at least 3x by the presence of TET proteins. Inset: Frequency distribution of TET activity as in Fig. 4c except bars are colored by the cutoff used to depict TET activity. (j) Comparison of passive (x-axis) and active (y-axis) demethylation rates inferred with high confidence. Red points in each panel represent CpGs from the chromatin context depicted in the plot. Chromatin states were used from a previous annotation55 and the key below and to the right represents ChIP signals represented in these states.

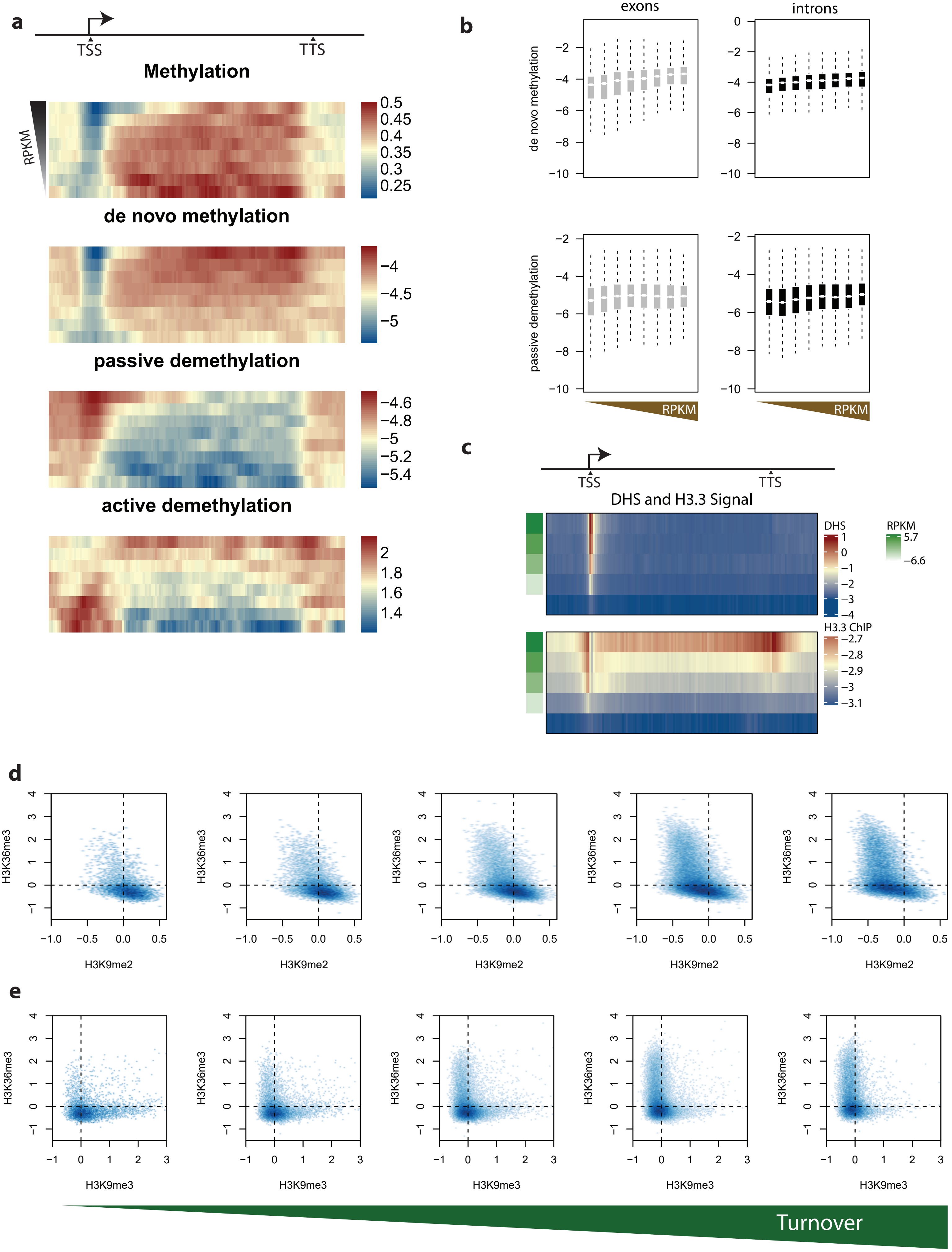

a
TSS
TTS
Methylation
RPKM
0.5
0.45
0.4
0.35
0.3
0.25
de novo methylation
-4
-4.5
-5
passive demethylation
-4.6
-4.8
-5
-5.2
-5.4
active demethylation
2
1.8
1.6
1.4
b
exons
introns
de novo methylation
passive demethylation
RPKM
c
DHS and H3.3 Signal
DHS
H3.3 ChIP
RPKM
5.7
−6.6
d
H3K36me3
H3K9me2
e
H3K36me3
H3K9me3
Turnover

Supplementary Figure 5. Methylation turnover at highly methylated CpGs correlates with transcriptional activity.
(a) Profiling of rates in gene bodies as described in Fig. 5b, except all genes in bins are at least 5kb in length to control for very small genes obscuring signal. Additionally, DHS peaks within genes were removed to exclude the possibility that trends were due to intragenic enhancers. Genes are additionally represented here by separation into 8 bins based on transcriptional output (RPKM). (b) Methylation rate as a function of exon or intron overlap (grey and white, respectively) and underlying transcriptional activity. Exons and introns were binned based on reads/kb within the respective regions. Transcriptional activity is increasing from left to right. k_{me} and k_{de} increase in both exons and introns concomitant with transcriptional activity, with a slightly higher increase in exons over introns. Boxplot elements are as described in Fig. 4d. (c) DHS and H3.3 ChIP signal as a function of transcriptional output. Genes were first binned on transcriptional output (RPKM) as in Fig. 5b. Genic bins were created such that the gene is represented by 100 equally sized bins with flanking 10kb regions divided into 25 bins each. DHS read counts were tallied as a function of bin position. Upper heatmap: While promoter accessibility increases as a function of transcriptional output, gene body accessibility is unchanged. Lower heatmap: H3.3 ChIP signal in murine ES cells[58] tallied in bins as above for DHS. Note scaling of genic and flanking signal with transcriptional output. (d) Relationship between H3K36me3, H3K9me2 and methylation turnover for highly methylated cytosines (>= 70%). Turnover bins increase from left to right, whereby K36me3 is highest in CpGs with higher turnover, while H3K9me2 decreases. (e) Same as in panel d except profiling H3K9me3 signal instead of H3K9me2 on the x-axis.

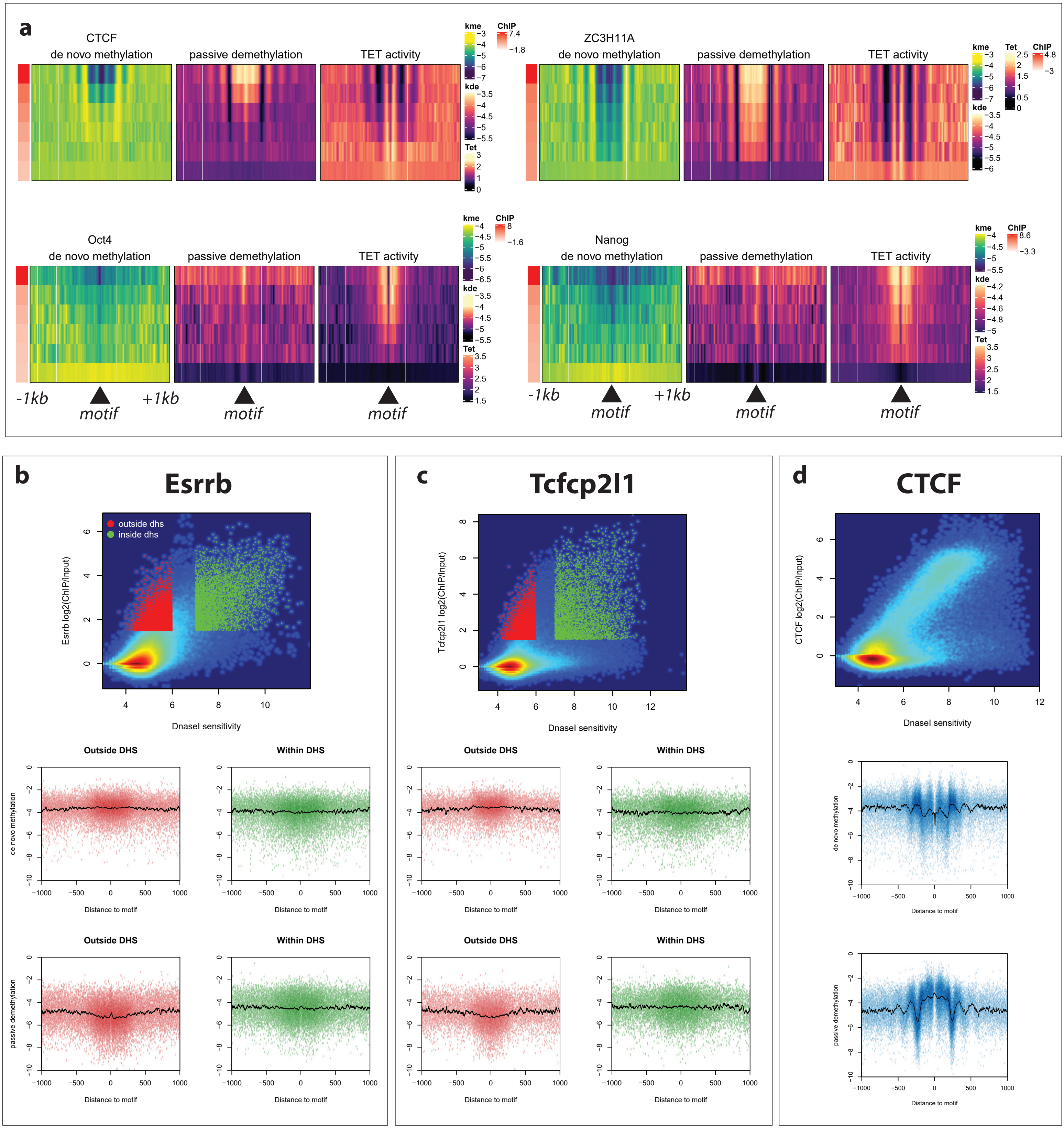

Supplementary Figure 6. TFs show variable effects on rates as a function of binding strength. (a) Influence of binding strength on rate patterns for CTCF, Oct4, ZC3H11A and Nanog. Each heatmap represents the average patterns of rates centered on a motif for the given factor. Rate of methylation is on the left, followed by passive demethylation and TET activity. Rows in the heatmaps represent bins of binding strength from ChIP data, represented by the red annotation column on the far right. Motifs were binned on binding strength, with strongest enrichment at the top. Each window consists of 2kb centered on the motif of interest. (b, c) Rates at CpGs surrounding TFs that show different pattern of k_{me} and k_{de}, namely a decrease in k_{de} coupled with a subtle increase in k_{me}. Scatterplots above depict binding strength (y-axis) as a function of DHS signal (x-axis). Binding sites with reduced DNAseI sensitivity are shown in red, while sites with increased DNAseI sensitivity are shown in green. Scatterplots below depict individual rate values for k_{me} and k_{de} at individual CpGs and the distance of that CpG to the center of the bound motif for that factor. Point colors represent the DHS distinction described above while the black line represents the running mean. (d) DHS signal as a function of binding for the top 10k represented CTCF motifs. Scatterplots below are as in panel b and c.
