# Supplementary material for: A genome-scale map of DNA methylation turnover identifies site-specific dependencies of DNMT and TET activity
Source: Nat Commun. 2020 May 29;11:2680. doi: 10.1038/s41467-020-16354-x (PMC7260214; doi:10.1038/s41467-020-16354-x)
Supplement: Supplementary file 3 — Reporting Summary [file 41467_2020_16354_MOESM3_ESM.pdf]

natureresearch

Corresponding author(s): Dirk Schübeler

Last updated by author(s): Mar 17, 2020

Reporting Summary

Nature Research wishes to improve the reproducibility of the work that we publish. This form provides structure for consistency and transparency in reporting. For further information on Nature Research policies, see Authors & Referees and the Editorial Policy Checklist .

Statistics

For all statistical analyses, confirm that the following items are present in the figure legend, table legend, main text, or Methods section.

n/a	Confirmed	
☐	☒	The exact sample size (*n*) for each experimental group/condition, given as a discrete number and unit of measurement
☐	☒	A statement on whether measurements were taken from distinct samples or whether the same sample was measured repeatedly
☐	☒	The statistical test(s) used AND whether they are one- or two-sided<br>*Only common tests should be described solely by name; describe more complex techniques in the Methods section.*
☐	☒	A description of all covariates tested
☐	☒	A description of any assumptions or corrections, such as tests of normality and adjustment for multiple comparisons
☐	☒	A full description of the statistical parameters including central tendency (e.g. means) or other basic estimates (e.g. regression coefficient) AND variation (e.g. standard deviation) or associated estimates of uncertainty (e.g. confidence intervals)
☐	☒	For null hypothesis testing, the test statistic (e.g. *F*, *t*, *r*) with confidence intervals, effect sizes, degrees of freedom and *P* value noted<br>*Give P values as exact values whenever suitable.*
☐	☒	For Bayesian analysis, information on the choice of priors and Markov chain Monte Carlo settings
☐	☒	For hierarchical and complex designs, identification of the appropriate level for tests and full reporting of outcomes
☐	☒	Estimates of effect sizes (e.g. Cohen's *d*, Pearson's *r*), indicating how they were calculated

Our web collection on statistics for biologists contains articles on many of the points above.

Software and code

Policy information about availability of computer code

Data collection	All publicly available data used in the study is referenced by accession number or the applicable manuscript directly.
Data analysis	All R packages used in the analysis and presentation of data are publicly available and referenced in the methods section. Functions relevant to each analysis are referenced in the methods with non-default parameters noted. Scripts used in generating individual figures as well as rate fitting are available upon request.

For manuscripts utilizing custom algorithms or software that are central to the research but not yet described in published literature, software must be made available to editors/reviewers. We strongly encourage code deposition in a community repository (e.g. GitHub). See the Nature Research guidelines for submitting code & software for further information.

Data

Policy information about availability of data

All manuscripts must include a data availability statement. This statement should provide the following information, where applicable:

- Accession codes, unique identifiers, or web links for publicly available datasets
- A list of figures that have associated raw data
- A description of any restrictions on data availability

Raw and processed sequencing data has been deposited in the Gene Expression Omnibus (GEO) database under the accession number GSE129470. All publicly available data sets used are referenced in the relevant methods section.
GEO link: https://www.ncbi.nlm.nih.gov/geo/query/acc.cgi?acc=GSE129470

Field-specific reporting

Please select the one below that is the best fit for your research. If you are not sure, read the appropriate sections before making your selection.

☒ Life sciences ☐ Behavioural & social sciences ☐ Ecological, evolutionary & environmental sciences

For a reference copy of the document with all sections, see nature.com/documents/nr-reporting-summary-flat.pdf

Life sciences study design

All studies must disclose on these points even when the disclosure is negative.

Sample size	No sample-size calculation was performed. Experiments were performed in triplicate to assess reproducibility of measurements.
Data exclusions	No data were excluded from the analyses.
Replication	Time course measurements were performed in triplicate to assure reproducibillity
Randomization	No randomization was performed
Blinding	Not applicable

Reporting for specific materials, systems and methods

We require information from authors about some types of materials, experimental systems and methods used in many studies. Here, indicate whether each material, system or method listed is relevant to your study. If you are not sure if a list item applies to your research, read the appropriate section before selecting a response.

Materials & experimental systems

n/a	Involved in the study	
☐	☒	Antibodies
☐	☒	Eukaryotic cell lines
☒	☐	Palaeontology
☒	☐	Animals and other organisms
☒	☐	Human research participants
☒	☐	Clinical data

Methods

n/a	Involved in the study	
☒	☐	ChIP-seq
☒	☐	Flow cytometry
☒	☐	MRI-based neuroimaging

Antibodies

Antibodies used	Slot blotting was carried out using an antibody against 5hmC (#39769, Active Motif) and 5mC (BI-MECY-1000, Eurogentec). Western blot antibodies used are DNMT3A (Novus 64B1446), DNMT3B (Imgenex IMG-184A), DNMT1 (AbCam ab188453), UHRF1 (MBL D289-3) and LAMINB1 (AbCam ab16048).
Validation	Antibodies were validated by mixing genomic DNA from unperturbed cell lines with discrete amounts of DNA from a published mouse stem cell line devoid of DNA methylation (see Supplementary Figure 1b). DNMT3 and DNMT1 antibodies were tested in a knockout background.

Eukaryotic cell lines

Policy information about cell lines

Cell line source(s)	ES cell lines conditionally deficient for Dnmt3a and Dnmt3b were derived by outgrowth of blastocyst embryos obtained by crossing mice doubly homozygous for floxed alleles of Dnmt3a and Dnmt3b (reference 50). The mouse strain was maintained on a C57BL6/J background.
Authentication	Mice were genotyped by PCR and ESCs derived from a homozygous clone. Conditional deletion of Dnmt3 alleles was carried out using TaqMan PCR as outlined in methods.
Mycoplasma contamination	Cell lines used in this study tested negative for mycoplasma contamination
Commonly misidentified lines (See ICLAC register)	*Name any commonly misidentified cell lines used in the study and provide a rationale for their use.*
